# Supplementary material for: Operando monitoring of ion activities in aqueous batteries with plasmonic fiber-optic sensors
Source: Nat Commun. 2022 Jan 27;13:547. doi: 10.1038/s41467-022-28267-y (PMC8795113; doi:10.1038/s41467-022-28267-y)
Supplement: Supplementary file 1 — Supplementary information [file 41467_2022_28267_MOESM1_ESM.pdf]

Supplementary information

Operando Monitoring of Ion Activities in Aqueous Batteries with Plasmonic Fiber-optic Sensors

Runlin Wang[1#], Haozhe Zhang[2#], Qiyu Liu[2#], Fu Liu[3#], Xile Han[1], Xiaoqing Liu[2], Kaiwei Li[1], Gaozhi Xiao[4], Jacques Albert[3], Xihong Lu[2]* & Tuan Guo[1]*

[1] Institute of Photonics Technology, Jinan University, Guangzhou 510632, People's Republic of China
[2] The Key Lab of Low-carbon Chem & Energy Conservation of Guangdong Province, School of Chemistry, Sun Yat-Sen University, Guangzhou 510275, People's Republic of China
[3] Department of Electronics, Carleton University, Ottawa K1S 5B6, Canada
[4] Advanced Electronics and Photonics Research Center, National Research Council of Canada, Ottawa K1A 0R6, Canada

[#] These authors contributed equally to this work.
* Correspondence: luxh6@mail.sysu.edu.cn (X.H. Lu); tuanguo@jnu.edu.cn (T. Guo)

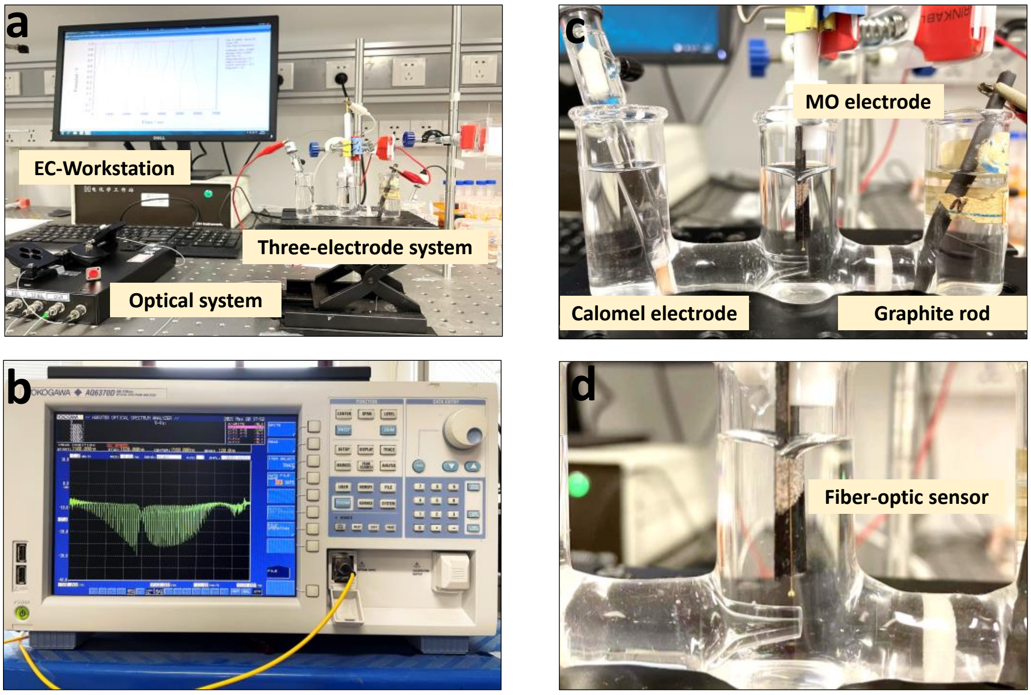

Figure S1 Instrumental photographs. **a** Whole experimental configuration, **b** Spectrum collected by the optical spectrum analyzer. **c** Three-electrode analysis system. **d** Local zoom view of working electrode: the fiber-optic sensor is attached to the surface of the electrode.

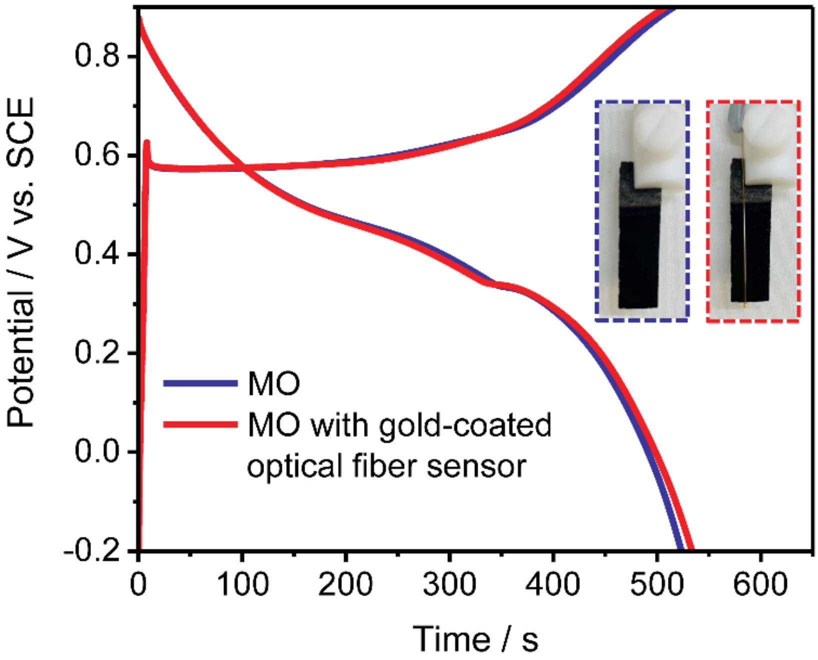

Figure S2 GCD curves at 1 mA cm^{-2} of MO electrode with/without the attached plasmonic fiber-optic sensor.

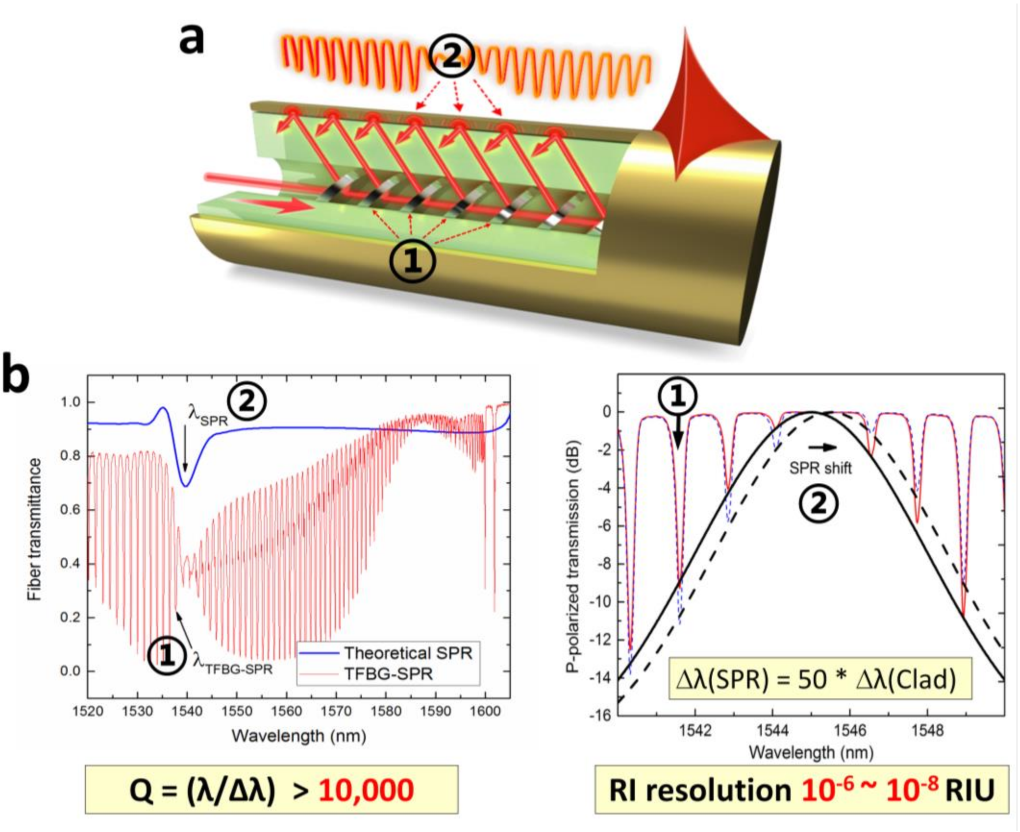

Figure S3 a Gold-coated TFBG with two hybrid resonances ①+② for SPR excitations. **B** Comparison between the best theoretical SPR response for 50 nm gold on silica in the Kretschmann-Raether configuration (thick blue line) and a measured TFBG-SPR spectrum with the same thickness of gold (thin red line). The arrows indicate the resonance to be followed in each case, in which the high Q-factor TFBG resonance (0.1 nm) provides a unique tool to measure small shifts of the Plasmon with high accuracy by transforming SPR wavelength shift into amplitude change.

Differing from the traditional bulk prism in the Kretschmann-Raether configuration, a tilted fiber grating assisted SPR sensor provides "TWO hybridized resonances ①+②" as **Figure S3a** shows. By tilting the grating fringes, the core-to-cladding resonant mechanism ① has the effect of providing a high-density narrowband spectral comb (tens to hundreds of narrowband cladding resonances with individual bandwidth ~0.1 nm and Q-factor at the level of 104) that overlap with the broader absorption (more than 10 nm wide in the best of cases) of the surface Plasmon ②. As in standard SPR, RI changes produce a shift of the SPR wavelength maximum. The hybridization of the grating resonances with that of the SPR provides a unique tool to measure small shifts of the SPR (due to RI changes) with high accuracy by measuring amplitude changes of the grating resonance located on the edge of the SPR as it shifts, as seen on **Figure S3b**.

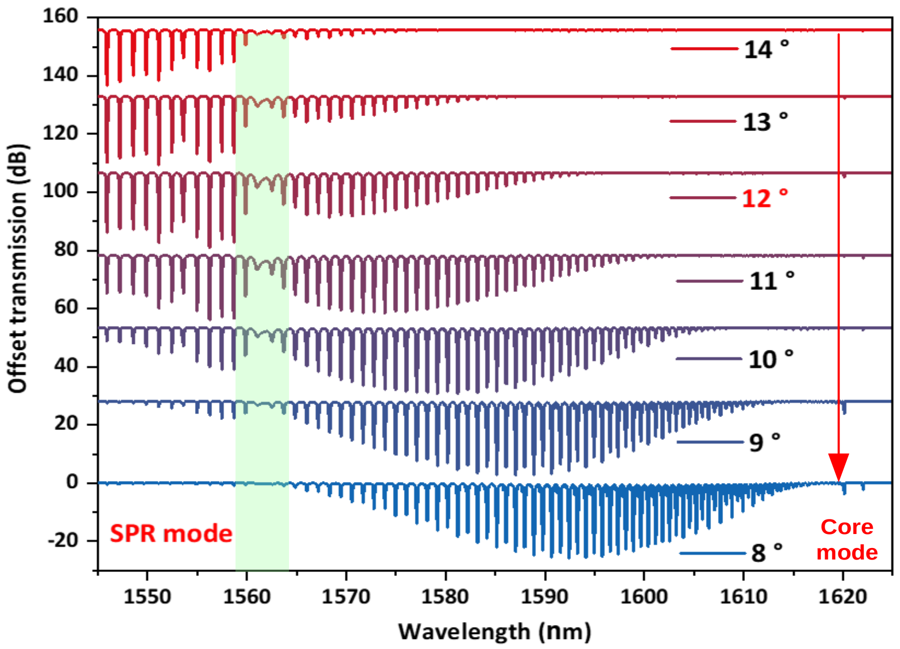

Figure S4 The simulation of transmission spectrum of the TFBG device was carried out by first calculating the vector mode fields and effective index of cladding modes as function of tilt angle and wavelength with a cylindrical finite difference mode solver, after which the corresponding spectra (P-polarized input core guided light) were achieved by using complex coupled-mode theory followed by a Runge-Kutta algorithm for the optical propagation through the grating. The properties used for the fiber were: core radius=4.1 um, cladding radius=62.5 um, cladding material of pure silica (SiO_2), and core material of germanium-doped silica with 0.0625 germanium/silicon ratio, Au thickness=50 nm (with accepted values for bulk Au thin film refractive indices, including wavelength dispersion).

The selection of the optimum tilting angle of fiber grating is determined by the refractive index of the electrolyte used in the Zn-ion batteries. The maximum sensitivity is obtained when the cladding modes have effective indices close to the index of the electrolyte which has been measured to be 1.33, with fluctuations of less than 0.02 during charging/discharging cycles. For a grating with a Bragg wavelength near 1620 nm, cladding modes with effective indices near 1.33 occur in the spectral region between 1560 and 1570 nm. In order to maximize the amplitudes of the cladding modes at these wavelengths, the tilt angle was selected based on simulations of TFBG spectra, as shown in **Figure S4**. They confirm that the maximum of the envelope of resonance amplitudes moves towards shorter wavelengths as the tilt angle increases and that the SPR location falls in the correct spectral window.

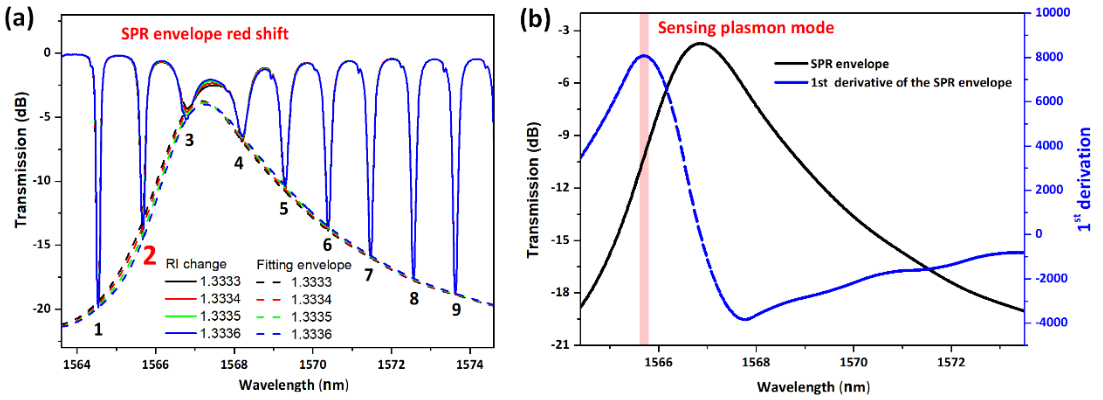

Figure S5 a The simulation of a 12° TFBG-SPR as a function of external refractive index (solid line). The corresponding fitting envelope of the attenuated cladding mode resonances covering the plasmon band is moving to longer wavelengths (dash line). **b** The fitted envelope of the SPR (black line) and its 1st derivative (blue line).

The resonance with the wavelength of 1565.7 nm is the first guided mode on the left of the most attenuated resonance. It is the most sensitive mode to external refractive index perturbation (hybrid plasmon guided mode) which is widely used for sensing. The corresponding simulation is shown in **Figure S5a**, in which all attenuated cladding modes covering the plasmon band are labeled from 1 to 10, and the corresponding fitted envelope is shifting to longer wavelength when external refractive index rises. If we perform a first derivative for the fitted envelope (**Figure S5b**), the cladding mode marked by number 2 shows the largest slope and therefore the most sensitive to RI changes, especially for very slight surface RI perturbations during the charging/ discharging process inside batteries.

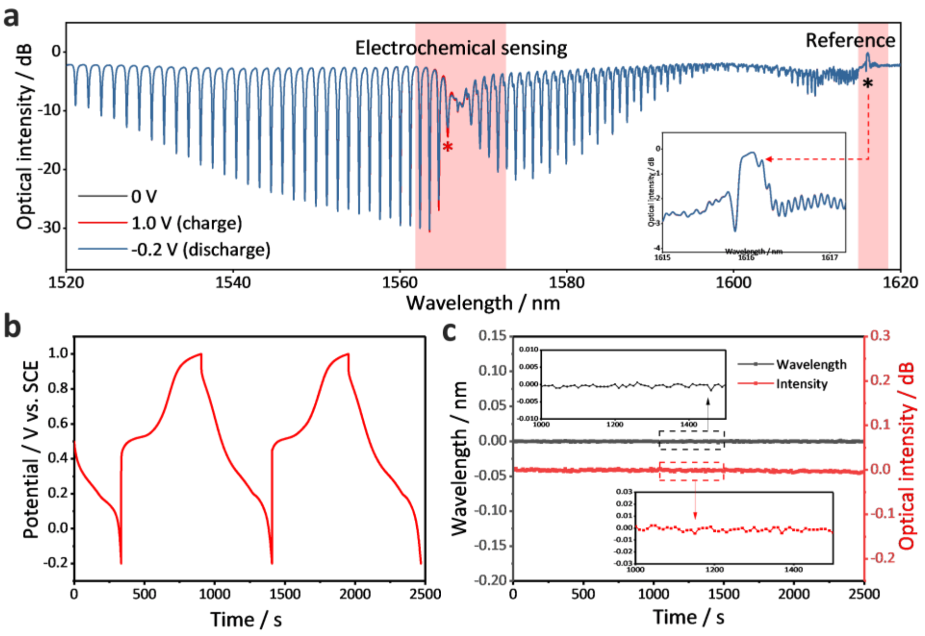

Figure S6 a Reflection spectral response of a plasmonic fiber-optic sensor for the different electronic polarization states of the electrode. **b** GCD curve at 1 mA cm^{-2} and **c** the stable response of core mode in wavelength and intensity during this process.

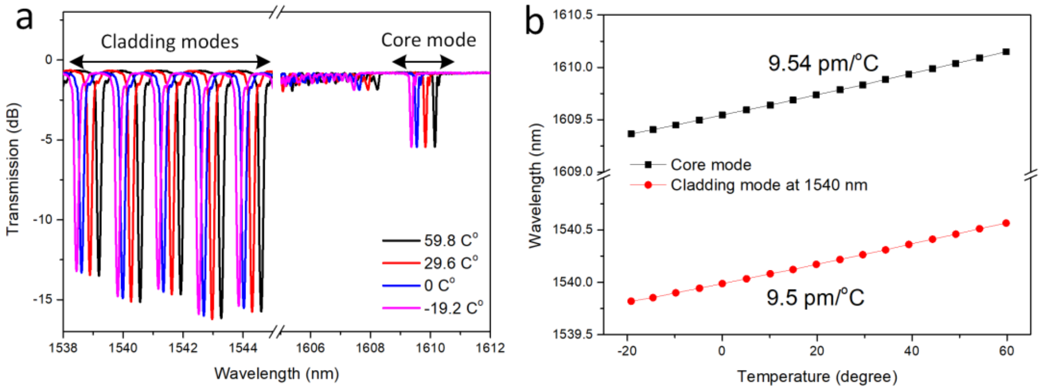

Figure S7 Experimental test of TFBG temperature responses: **a** Transmission spectra of 12° TFBG as a function of external temperature; **b** the temperature sensitivity of core mode (reference mode) and high order cladding mode.

As for the cross-sensitivity of temperature: it will be perfectly removed temperature crosstalk by considering the RELATIVE wavelength shift of cladding mode to core mode (core mode is independent of external RI perturbation) because they are showing the "same" temperature sensitivity, shown in the **Figure S7**. In practical application inside batteries, the temperature variation will be compensated in real time during the data collection of both cladding mode and core mode, by a data processing correction of the cladding mode shifts from that of the core mode shift.

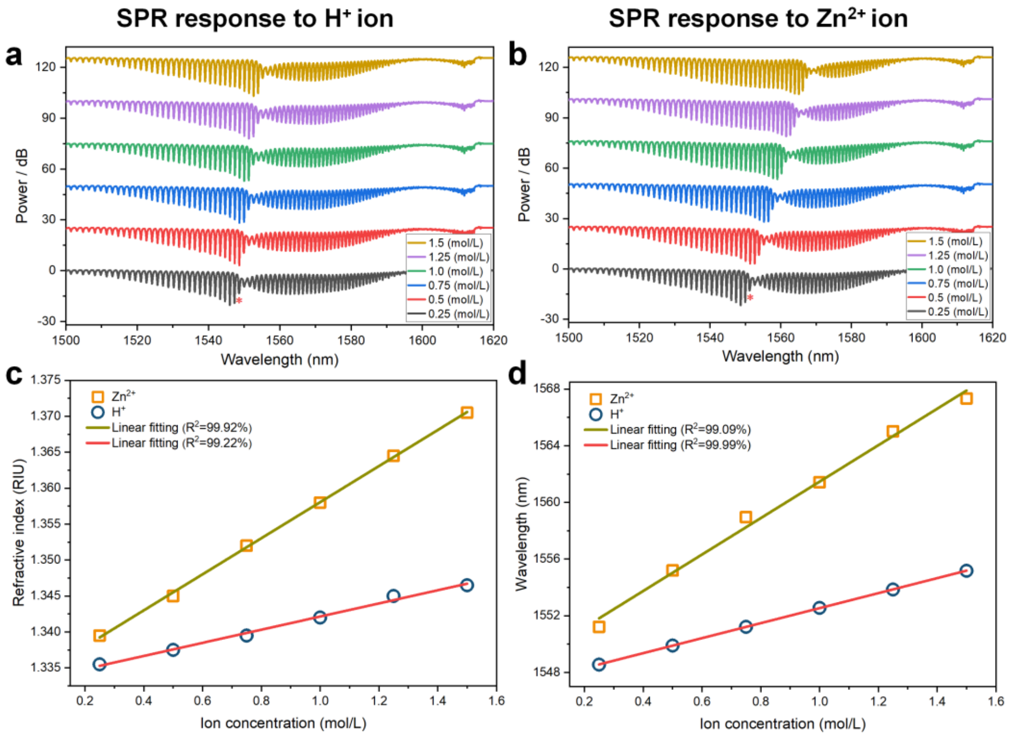

Figure S8 Experimental spectral response of TFBG-SPR as a function of H^+ (a) and Zn^{2+} (b) concentration. The Zn^{2+} ion liquid is obtained by the process where 7.2, 14.4, 21.5, 28.7, 35.9 and 43.1 g of zinc sulfate heptahydrate were dissolved in 100 mL of deionized water, respectively, and stirred by magnetic force until dissolved to obtain 0.25, 0.50, 0.75, 1.00, 1.25 and 1.50 M zinc sulfate solutions. The same series of sulfuric acid solutions were prepared with concentrated sulfuric acid (95.0%-98.0%). (c) the refractive index of H^+ (Zn^{2+}) ion concentration tested by commercial abbe refractometer (Shanghai LICHEN-BX Instrument Technology, WAY-2WAJ, resolution: ±0.0002 RIU) at the constant temperature of 24 ^{o}C, and (d) the corresponding SPR (attenuated cladding mode family in (a) and (b)) wavelength position.

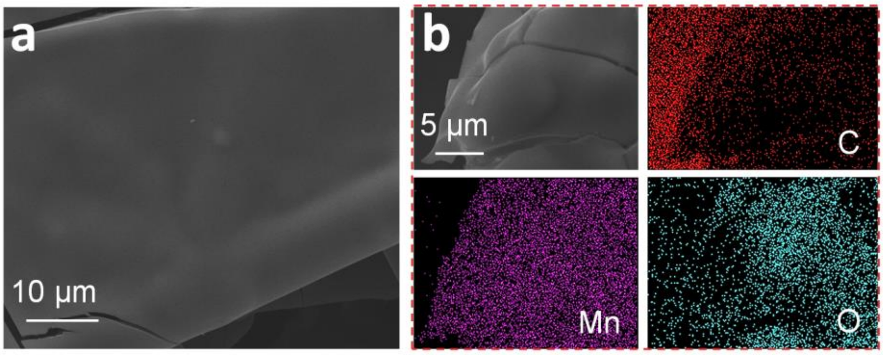

Figure S9 a SEM images and **b** mapping of MO electrode.

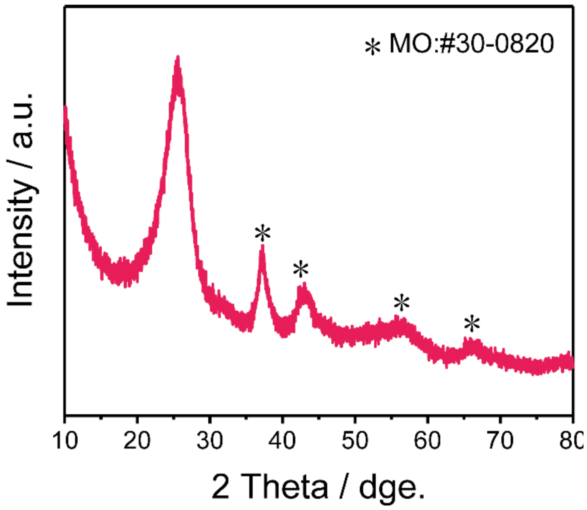

Figure S10 XRD pattern of MO electrode (the peak near 26° corresponding to the carbon substrate).

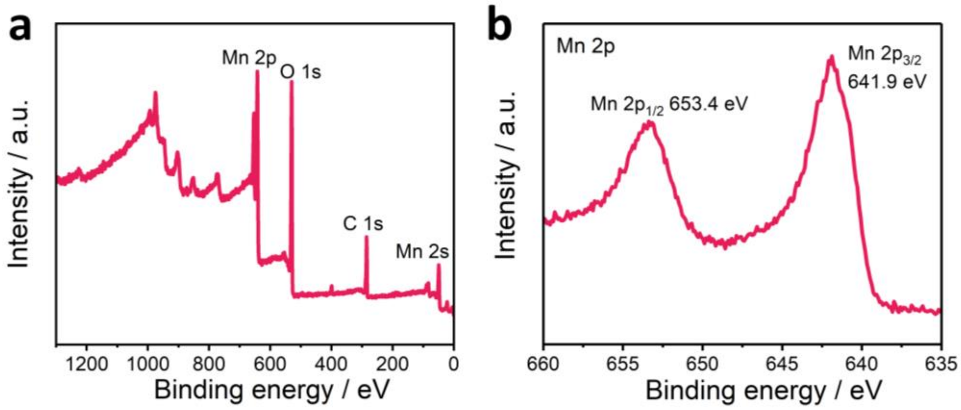

Figure S11 a XPS survey spectra. The peaks corresponding to N 1s (appearing at 399.5 eV) is caused by the use of nitrate residues during the synthesis of the material. **b** Mn 2p XPS profiles of MO electrode, in which two peaks located at the binding energy of

641.9 eV and 653.4 eV correspond well to the $Mn2p_{3/2}$ and $Mn2p_{1/2}$ peaks of Mn^{4+}.

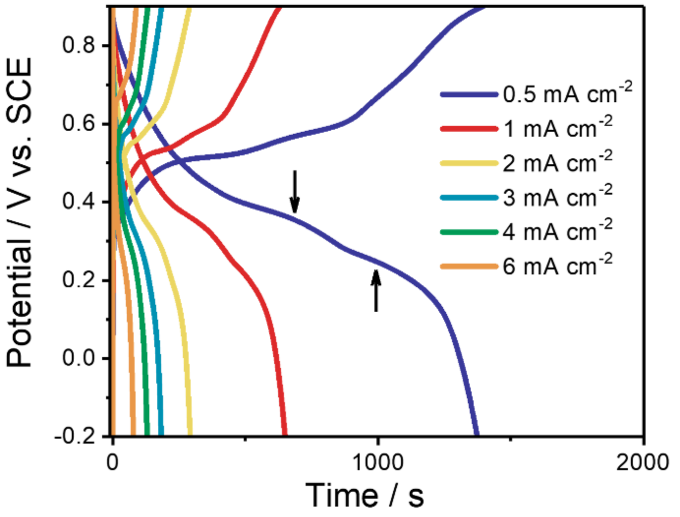

Figure S12 GCD curves of MO electrode at various current densities.

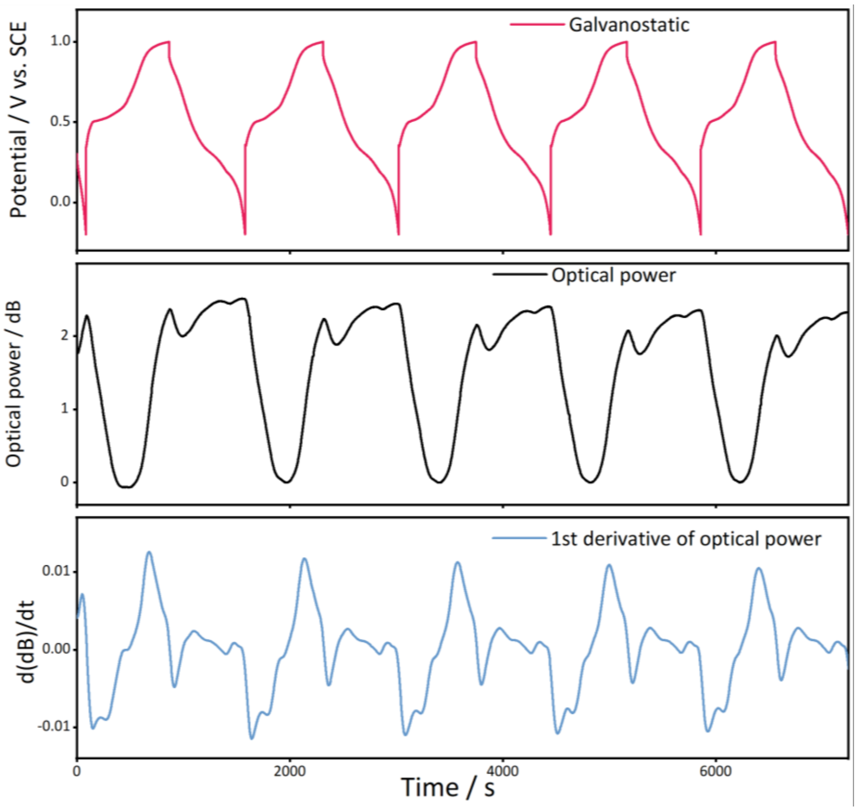

Figure S13 GCD curve at 1 mA cm^{-2} (red), SPR intensity curve (black) and differential of light intensity (blue) of MO electrode.

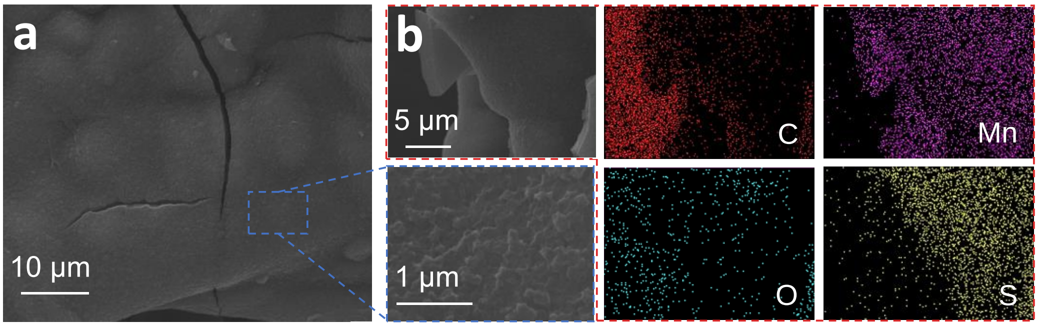

Figure S14 a SEM images and **b** zoomed mapping of MO@PEDOT electrode.

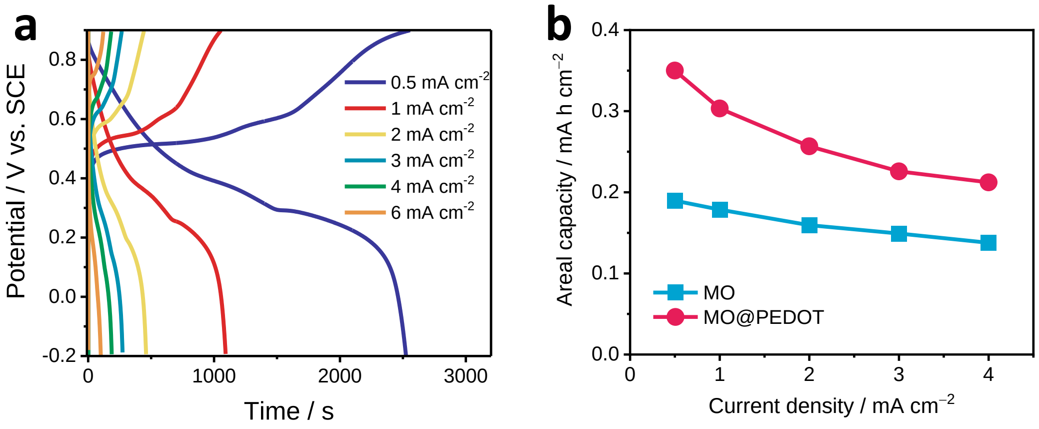

Figure S15 a GCD curves of MO@PEDOT electrode at various current densities. **b** Areal capacity as a function of the current densities of MO and MO@PEDOT electrodes.

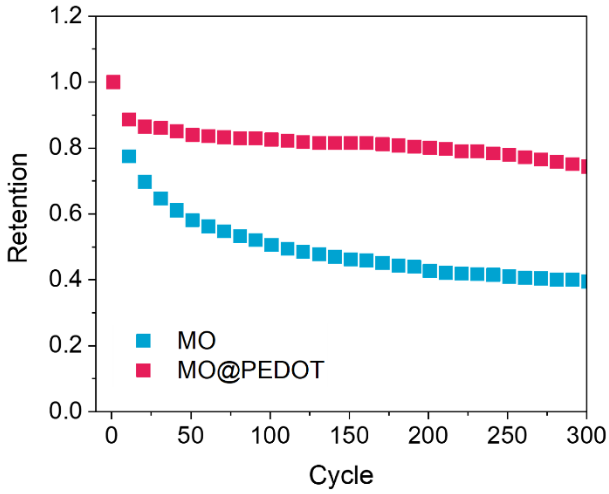

Figure S16 Cyclic stability at 15 mA cm^{-2} for 300 cycles of the Zn-ion batteries based on the MO or MO@PEDOT electrodes. The batteries are assembled by cathode, Zn foil anode and 1 M $ZnSO_4$+0.4 M $MnSO_4$ aqueous electrolyte. The voltage window is 0.8-1.9 V. The MO@PEDOT//Zn battery retains 75% of the original capacity, while the MO//Zn battery only retains 40%.

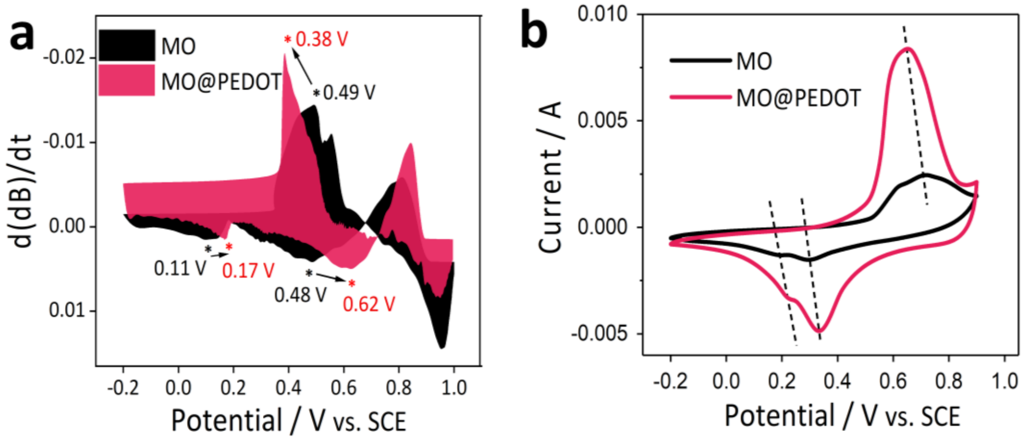

Figure S17 Spotting voltage-resolved relationship curve of MO and MO@PEDOT electrodes. **a** Derivative of the SPR intensity P'/V and **b** the corresponding CV curve at 1 mV s^{-1}.

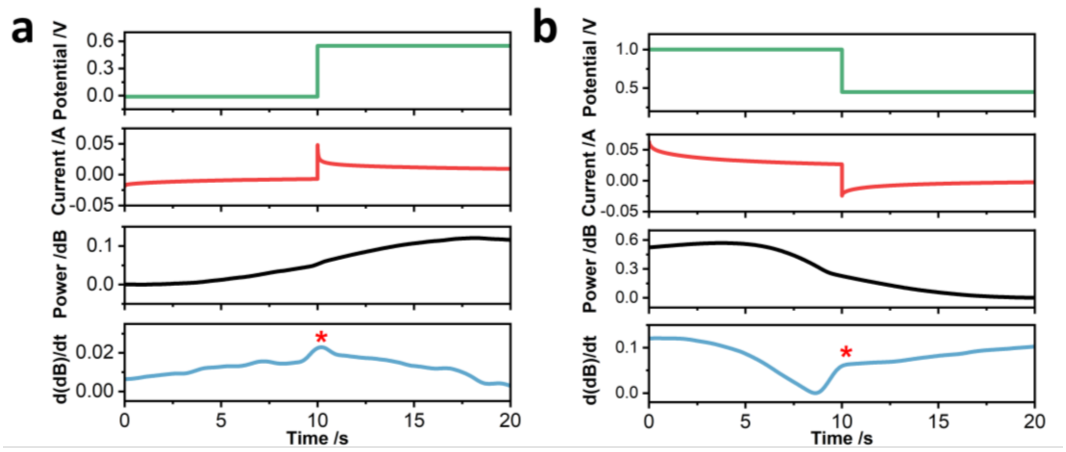

Figure S18 Potential versus time curve (green), PSCA curve (red), SPR power curve (black) and differential of light power (blue) of MO@PEDOT electrode. The two potential stepping models (**a** from -0.1 V to 0.55 V and **b** from 1 V to 0.45 V) are carried out to research the ion kinetic of MO@PEDOT electrode at charging and discharging states, respectively. To be specific, the tests are carried out using a MO@PEDOT electrode which is in a full-ion-intercalation-state after being stabilized by two charging and discharging cycles under GCD tests. During the test, the potential is changed after a standing time of 10 s. Obviously, the direction of the current is reversed at the potential stepping point. Meanwhile, the SPR power level increases at ion deintercalation potential (0.55 V) while decreases at ion intercalation potential (0.45 V), which indicates the change of ion concentration at electrolyte-electrode interface. Moreover, both d(dB)/dt curves possess an obvious peak (red star), demonstrating a sudden increase in ion transfer rate at the stepping points.

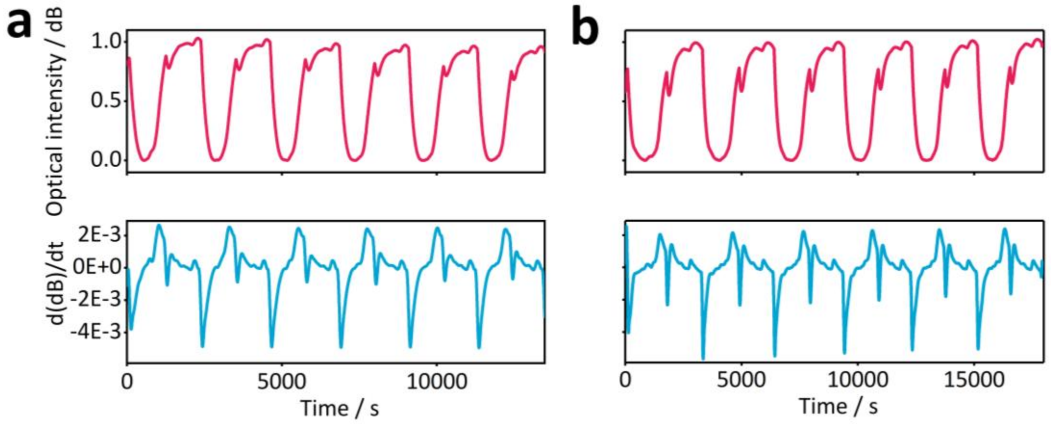

Figure S19 Normalized SPR intensity curve (red) and differential of optical intensity (blue) at the discharge stage of **a** MO and **b** MO@PEDOT electrodes.

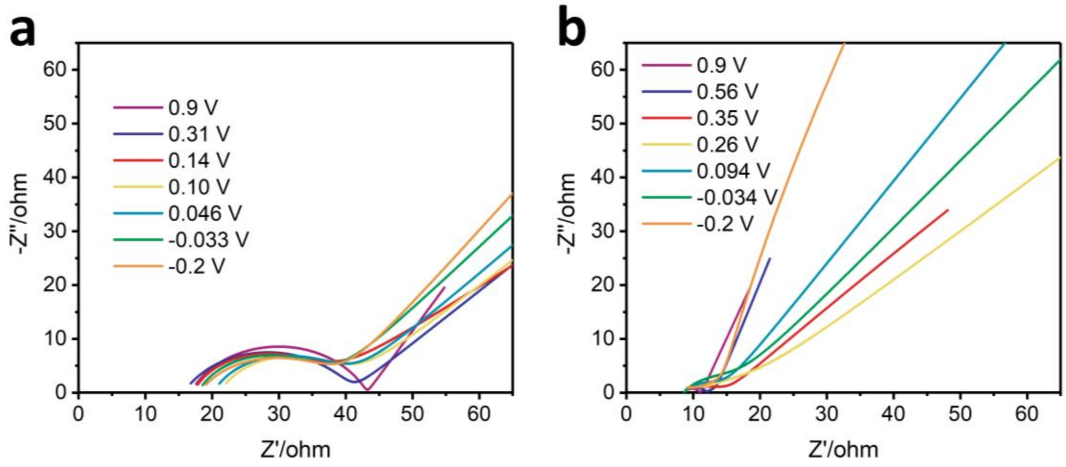

Figure S20 Nyquist plots of **a** MO and **b** MO@PEDOT electrodes at different potentials during discharge process.

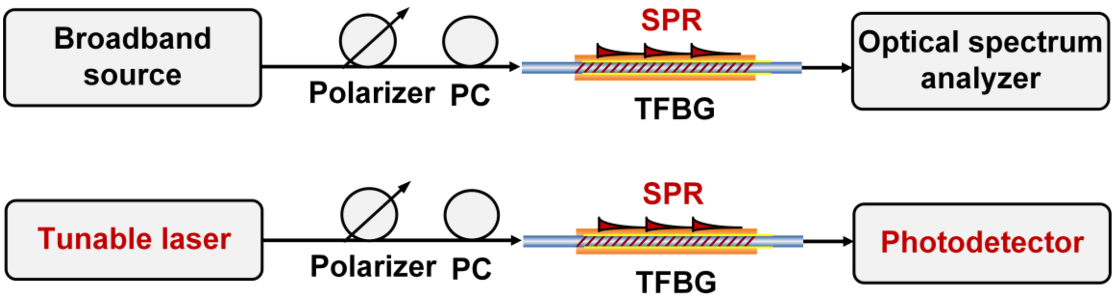

Figure S21 Scheme of the two typical implementations of optical spectral (up) and optical power (bottom) interrogation of SPR optical fiber sensor.

To realize a portable and compact instrumentation for in-field battery measurements ranging from electric cars to power plants, we can use a real-time interrogation scheme by replacing the wavelength interrogation by optical power detection, as **Figure S**** shown. In this case, a tunable laser can be used as a source instead of broadband light source, together with a photodiode as detector and an analog-to-digital converter to obtain the desired data (to replace the optical spectrum analyzer). The function of the tunable laser is matching the wavelength of the most sensitive SPR mode of the TFBG, so once that the sensor is characterized it can be replaced by a common laser (for example, a compact VCSEL). This technique relies on the principle of edge filtering so that the optical power change is produced as a result of the wavelength shift of the mode with respect to the fixed wavelength of the laser source.

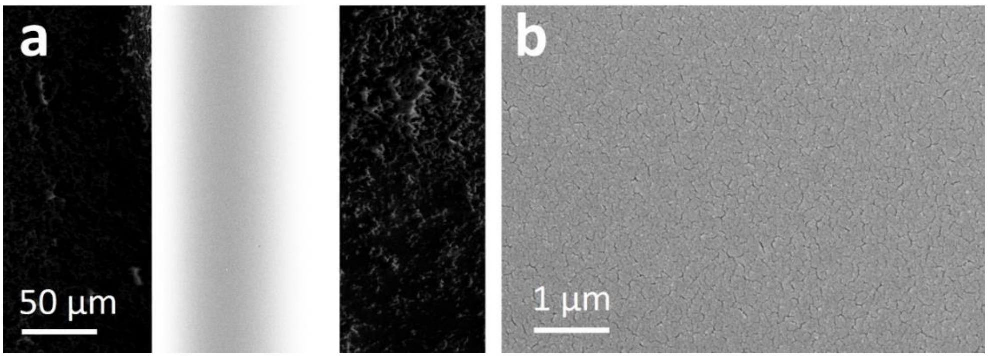

Figure S22 SEM images of **a** plasmonic fiber-optic sensing probe and **b** gold film on the fiber surface.

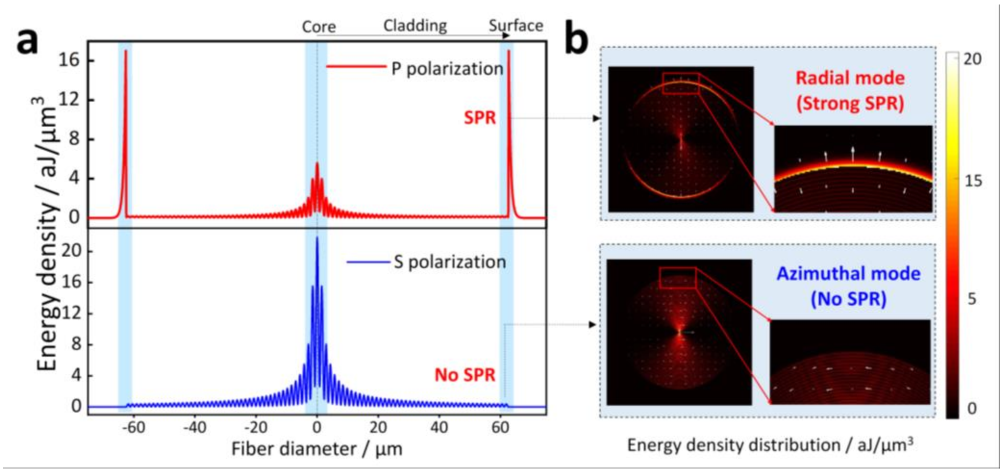

Figure S23 a Simulated radial energy density distribution of plasmonic fiber-optic sensor under P-polarized incident light (red) and S-polarized incident light (blue). **b** Simulated electric mode field profiles for high-order cladding modes with two orthogonal polarizations (P- and S-polarizations, respectively).
